# 

**Published:** 1887-05

**Authors:** 


### BOOK NOTICES.

CAPTAIN GLAZIER AND HIS LAKE.—This is the title of a pamphlet of some sixty pages, by Henry D. Harrower, devoted to an exposition of the false claims of one Captain Glazier, as the one simon-pure-original-jacobs discoverer of the *ne-plus-ultra* source of the Father of Waters, which would have been all very well, if it had not been discovered long before by other well-known explorers, among whom the name of Schoolcraft will be familiar to our readers, concerning whose narrative, published in 1834, and Captain Glazier's fifty years later, there is a most remarkable resemblance of incident and phraseology.

We are heartily rejoiced that this *cheap-john* pretender has been so thoroughly exposed, that the fame which he sought at the expense of his betters, has been whittled down to a fine point of contempt. After this *harrowing*, we don't see what there is open to this Falstaffian captain of an explorer, more suited to his genius than a hand-organ and a monkey.

We offer our condolence to the people and Geographical Societies which have bestowed upon Capt. Glazier the honors of a great discoverer. There is an anecdote of the celebrated Dr. Samuel Johnson, and one of the old time *glazier* family, which would not be altogether inaptly applied to this one. The Doctor was taking one of his customary walks about town, when he saw a common sort of person deliberately break a window pane of a dwelling. "You rascal! what do you mean by that?" said the Doctor, addressing him. "Im a *glazier*, sir, an' I must live," replied the fellow. "And I'm a *surgeon*, and *I* must live," responded the Doctor, as he brought his cane down in no gentle way upon the glazier's cranium.

But we opine that in spite of the stripping off of the lion's skin, the Captain will still insist upon spelling his name with a capital G and indulging himself in tales of ction.

See *Educational Reporter*, (Extra), Iverson, Blakeman & Co., New York Publishers.

NATURAL LAW IN THE BUSINESS WORLD.—This is a timely volume by Henry Wood, whose aim is to show that labor and trades organizations are opposed to the best interests of the laboring and trades classes, inasmuch as they are based upon a false theory of conflict between labor and capital, whereas, in a healthy and well-regulated community, there can be no conflict between elements which mutually depend upon one another for any advantageous outcome to either.

They are also unnatural as tending to interrupt the otherwise steady flow of supply and demand, which are the life of the State. Capital unemployed is like a field going to waste for want of cultivation ; and idle workmen are like the stubble that stands in the place of a last year's crop, but promises nothing for the future.

The above work seems to cover the whole argument with plainly distinguished lines between the natural and practical and the artificial and impracticable.

Published by Charles T. Dillingham, New York.

HORTICULTURAL ART JOURNAL, Rochester.— American News Company general agents. The April number of this superbly illustrated monthly is before us, and fully sustains its well-earned reputation. The illustrations are of apples and roses.

JAMES VICK, SEEDSMAN.—Our thanks are due to James Vick, of Rochester, N. Y., the famous seedsman and publisher of Vick's illustrated *Floral Guide*, for an ample supply of flower seeds suited to the floral garden.

We are able to recommend Mr. Vick's establishment as one of the most reliable of its kind in this country.

